# Chemical Profiling and In Vitro Evaluation of Bioactive Properties of *Evernia prunastri* Extract: Implications for Therapeutic Applications

**DOI:** 10.3390/plants14040583

**Published:** 2025-02-14

**Authors:** Dejan Stojković, Jelena Živković, Stefani Bolevich, Sergey Bolevich, Gokhan Zengin, Uroš Gašić, Marina Soković

**Affiliations:** 1 Institute for Biological Research “Siniša Stanković”—National Institute of the Republic of Serbia, University of Belgrade, Bulevar despota Stefana 142, 11108 Belgrade, Serbia; dejanbio@ibiss.bg.ac.rs (D.S.); uros.gasic@ibiss.bg.ac.rs (U.G.); 2 Institute for Medicinal Plants Research “Dr. Josif Pancic”, Tadeusa Koscuska 1, 11000 Belgrade, Serbia; 3 Department of Pathologic Physiology, First Moscow State Medical University I.M. Sechenov (Sechenov University), 119991 Moscow, Russia; alistra555@mail.ru (S.B.); bolevich2011@yandex.ru (S.B.); 4 Department of Biology, Science Faculty, Selcuk University, Konya 42130, Turkey; biyologzengin@gmail.com

**Keywords:** *Evernia prunastri*, phenolics, enzyme inhibition, antioxidant activity, antimicrobial activity, cookies

## Abstract

*Evernia prunastri* (L.) Ach. (Parmeliaceae), an edible lichen commonly known as oakmoss, was traditionally used by Egyptians to make bread. In this study, the ethyl-acetate (EtOAc) extract of *E. prunastri* was investigated for its potential therapeutic applications in *diabetes mellitus*, Alzheimer’s and Parkinson’s diseases, oxidative stress, and bacterial infections. The extract exhibited significant in vitro enzyme inhibition activities, including anti-amylase and anti-glucosidase activities linked to diabetes and anti-cholinesterase and anti-tyrosinase activities associated with Alzheimer’s and Parkinson’s diseases. The antioxidant activity was evaluated through multiple assays, including free radical scavenging (DPPH and ABTS), reducing power (CUPRAC and FRAP), metal chelation, and phosphomolybdenum methods, demonstrating strong oxidative stress relief potential. The antibacterial properties were also confirmed through antibacterial testing, showing efficacy against a range of bacterial strains. Total phenolic and flavonoid contents were quantified, while the chemical profile of the EtOAc extract was determined by LC-HRMS/MS analysis. The chemical composition was predominantly characterized by depsides (evernic acid and atranorin), phenolic acids (orsellinic acid), and dibenzofurans, revealing a diverse array of bioactive secondary metabolites. The extract demonstrated a broad spectrum of biological activities, including enzyme inhibition, antioxidant effects, and antibacterial properties. This study highlights the potential of *E. prunastri* as a functional food, providing a rich source of bioactive compounds with numerous health-promoting effects, and it suggests its relevance in therapeutic applications for chronic diseases such as diabetes, Alzheimer’s, and bacterial infections.

## 1. Introduction

*Evernia prunastri* (L.) Ach. (Parmeliaceae), also known as oakmoss, can be found in temperate mountainous forests across the Northern Hemisphere, including regions of France, Portugal, Spain, North America, and the greater part of Central Europe. Besides its usage in modern perfumery, it has been used by Egyptians for making bread [1]. Lichen-forming fungi are symbiotic organisms that establish associations with green algae, cyanobacteria, or a combination of both [2]. They synthesize numerous natural products, many of which possess biological activities [3]. Whole lichen extracts have shown antimicrobial, anti-inflammatory, analgesic, or cytotoxic activity [3], rendering lichens interesting organisms for pharmaceutical applications [4] as supplements, enlightening the traditional use of edible species as foods with many beneficial health effects. One of the ecological roles of lichen compounds is to protect them against damage from from sunlight [5]. The main research hypothesis of this study was that *E. prunastri* extract possesses a diverse range of bioactive properties that could have therapeutic potential in treating conditions such as diabetes, Alzheimer’s disease, oxidative stress, and bacterial infections.

*Diabetes mellitus* is a chronic metabolic disorder characterized by elevated blood glucose levels, which result from insufficient or ineffective insulin production. This condition leads to disruptions in the metabolism of carbohydrates, proteins, and lipids [6]. A practical approach to decrease postprandial hyperglycemia is to prevent the absorption of carbohydrates after food intake. Therefore, α-amylase and α-glucosidase inhibitors may help to reduce postprandial hyperglycemia by inhibiting the enzymatic hydrolysis of carbohydrates, and hence, they may delay the absorption of glucose. Several synthetic drugs, including acarbose, voglibose, and miglitol, are generally applied as enzyme inhibitors in the treatment of type 2 diabetes [7]. However, these inhibitors are reported to cause several side effects, such as abdominal distention, flatulence, meteorism, and possibly diarrhoea [8]. Efforts have been made to find natural and safer α-amylase and α-glucosidase inhibitors.

Alzheimer’s disease (AD) is a progressive neurological disease connected with reductions in acetylcholine (ACh) and butyrylcholin (BCh) levels in the cortex and hippocampus in the brain [9]. Inhibition of cholinesterase enzymes, which break down ACh and BCh, could be a possible therapeutic approach for AD patients [9]. Parkinson’s disease (PD) is also a progressive neurodegenerative disease connected with dopamine deficiency in the brain. The present treatment includes the general use of L-DOPA and dopamine agonists [10]. Moreover, numerous studies have suggested that tyrosinase may be involved in neuromelanin production and neuron damage characteristic of PD, highlighting tyrosinase inhibitors as potential therapeutic agents [11].

Oxidative stress is a condition of the body that occurs when the body is unable to neutralize free radicals. Oxidative stress is accompanied by malaise, lethargy, rapid aging of the organism, insomnia, rapid heartbeat, etc. Antioxidants are substances that prevent the formation of new free radicals as well as destroying already existing one, and they also repair damage in cells. Therefore, identifying novel natural sources of antioxidants capable of neutralizing free radicals is a primary focus in the field of disease prevention [12].

Although there are effective methods and reliable drugs to treat microbial infections, some bacteria have developed mechanisms to overcome the effects of commercial antimicrobials, most often due to their misuse. Therefore, the need for novel natural medicines to combat bacteria has increased over recent decades and represents a significant goal worldwide [13,14].

This paper aimed to describe the potential biological properties of ethyl-acetate (EtOAc) extract of *E. prunastri* collected from the mountain region in south Serbia. It aimed to determine whether this edible lichen could be described as a functional food with different pharmacological effects. In vitro tests were performed to link the potential therapeutic effects of the lichen with important health-related disorders such as *diabetes mellitus*, Alzheimer’s and Parkinson’s diseases, oxidative stress, and bacterial infections.

The novelty of our study lies in the use of EtOAc extract, which has not been extensively explored in previous investigations of *E. prunastri*. Most existing studies focus on extracts obtained through different solvents. By using EtOAc, we aimed to target a distinct range of bioactive compounds that may not be effectively extracted through other solvents, potentially offering unique biological properties. Our study integrates comprehensive enzyme inhibition assays related to diabetes and neurodegenerative diseases, alongside antioxidant and antibacterial evaluations, further highlighting the diverse pharmacological potential of EtOAc extract.

## 2. Results and Discussion

### 2.1. Quantification of Total Phenolics and Flavonoids in the Sample and Metabolomics

The yield of dry extract of *E. prunastri* was 19.54%. The data presented in Table 1 illustrate the total phenolic and flavonoid contents, as well as the concentrations of specific metabolites in the *E. prunastri* EtOAc extract. The extract demonstrated phenolic levels of 22.11 mg gallic acid equivalent (GAE) per gram of dry weight (dw) and flavonoid levels of 13.64 mg rutin equivalent (RE) per gram of dw, indicating potential antioxidant activity. These results are generally lower than or comparable to those reported by other researchers. Namely, Studzinska-Stroka et al. [15] examined *E. prunastri* extracts of different polarities obtained by ultrasonic extraction four times at a temperature of 40 °C and reported TPC values ranging from 80 to 150 mg GAE/g dw. According to their study, extracts with the lowest polarity were richer in polyphenolic compounds. In another work, authors investigated TPC in water, methanolic, and acetone *E. prunastri* extracts and noted values ranging from 6.20 to 13.17 mg GAE/g dw [16], which are similar to our results.

The compounds were identified based on the observed mass spectra and compared with data in the literature. The LC-HRMS/MS analysis of the EtOAc extract of *E. prunastri* identified 20 major secondary metabolites, including phenolic acids, depsidones, dibenzofurans, and lichen-specific compounds. Details of the identified compounds, including their theoretical and observed mass-to-charge ratios, molecular formulas, absolute errors in parts per million (ppm), and retention times (Rt) in the positive-ion mode in ESI, are presented in Table 1.

The retention times (Rt) ranged from 5.58 to 10.74 min, indicating the presence of both early- and late-eluting compounds with varying polarities. The molecular formulas suggest a predominance of oxygenated structures, consistent with known lichen metabolites. Mass spectrometric analysis provided high-resolution exact masses with low mass errors (Δ ppm generally within ±2), confirming accurate molecular identification.

Almost 85% of lichen secondary metabolites are unique to the lichenized state and are not found in higher plants or free-living fungi [17]. In our study, several key compounds were identified. Orsellinic acid (Rt 5.58 min, C_8_H_7_O_4_^−^), a known lichen-derived phenolic acid, was found at 167 *m*/*z*. Salazinic acid (Rt 7.14 min, C_18_H_11_O_10_^−^), a depsidone with potential antioxidant and antimicrobial activities, was found at 387 *m*/*z*, typical for this compound class. Evernic acid (Rt 9.12 min, C_17_H_15_O_7_^−^) and atranorin (Rt 10.43 min, C_19_H_17_O_8_^−^) are well-known depsides with significant antimicrobial and anti-inflammatory properties [18]. Physodic acid (Rt 9.73 min, C_26_H_29_O_8_^−^) and hydroxyphysodic acid (Rt 9.23 min, C_26_H_29_O_9_^−^) belong to the physodic-acid-type lichen metabolites with reported cytotoxic and antioxidant effects [19]. These depsidones were previously found in other lichen species, with *Hypogymnia physodes* as one of the most abundant sources [20]. Compound 8 was identified as parmasidone B based on previous data found in the literature about an investigation of the lichen *Parmotrema tsavoense* [21].

The MS^2^ fragmentation data provide additional structural confirmation. Many compounds exhibit neutral losses typical for lichen phenolics (e.g., loss of CO_2_ or CH_3_), and the base peaks in the MS^2^ spectra align with reported fragmentation patterns from previous studies. Some compounds, such as chloroevernic acid (Rt 9.23 min, C_17_H_14_ClO_7_^−^) and chloroatranorin (Rt 10.74 min, C_19_H_16_ClO_8_^−^), feature halogenation, which may enhance their bioactivity, particularly against microbial pathogens. The fragmentation pattern of compound 15 was also in accordance with previously published data [22], and it was identified as the phenolic compound cristiferid B. This analysis highlights the diverse metabolic profile of *E. prunastri*, including several bioactive lichen secondary metabolites with antioxidant, antimicrobial, and potentially cytotoxic properties. Further studies are needed to explore their functional roles and applications.

**Figure 1:**
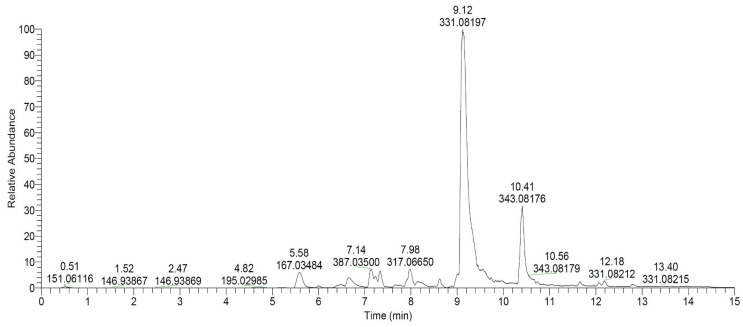
Chromatogram of the EtOAc extract of *E. prunastri*, showing the relative abundance of compounds detected across retention times (Rt, min) with annotated *m*/*z* values for major peaks.

The chromatogram of the *E. prunastri* EtOAc extract revealed a diverse chemical composition with a well-defined separation of compounds (Figure 1). The chromatogram highlighted several significant peaks, with the most prominent one observed at Rt 9.12 min, corresponding to an *m*/*z* value of 331, which represents evernic acid. This compound is a well-known lichen metabolite with significant bioactivity. Another major peak appeared at Rt 10.41 min with an *m*/*z* value of 343, corresponding to atranorin.

The chromatogram demonstrated a stable baseline with no significant drift, reflecting the high quality of the separation and detection system. The rich chemical diversity observed in the *E. prunastri* EtOAc extract, dominated by evernic acid and atranorin, highlights the potential bioactive properties of the extract, which likely stem from the combined effects of phenolic acids, depsides, and dibenzofurans.

According to our knowledge, several research groups have previously recorded the phytochemical composition of *E. prunastri* extracts. According to these studies, *E. prunastri* was reported to contain evernic acid, atranorin, methyl lecanorate, and chloroatranorin [23,24]. The specific profile of the compounds listed in the Table 1 has not previously been reported from *E. prunastri*. In our study, the specific composition of the extract might have been influenced by both the extraction system (solvent) and the geographical location of the sample collection. Different solvents can selectively extract compounds with varying polarities, which can result in differences in the composition of the extracts. This aspect has been documented in a previous study [25], which highlights that the solvent choice significantly impacts the yield and composition of bioactive compounds. Geographical location also contributes to the chemical diversity of plant extracts. Variations in climate, soil composition, altitude, and other environmental factors can lead to differences in metabolism, thereby affecting the chemical composition of the extracts. This phenomenon has been observed in other studies on natural extracts [26], where samples from different regions exhibited significant variations in their specialized metabolite profiles.

### 2.2. Enzyme Inhibition

Taking into account the importance of specific enzymes in the etiology and pathogenesis of neurodegenerative diseases and type 2 diabetes mellitus, as well as the recently increased incidence of the mentioned diseases, we studied the effect of the EtOAc extract of *E. prunastri* on the activities of the following enzymes: AChE, BChE, tyrosinase, amylase, and glucosidase.

The enzyme inhibitory profile of the *E. prunastri* EtOAc extract, as presented in Table 2, highlights its potential for applications in neuroprotection and metabolic regulation. Specifically, the extract showed mild inhibition of cholinesterasease with IC_50_ values of 1.26 mg/mL (for AChE) and 1.70 mg/mL (for BChE) as compared with galanthamine. These levels suggest that while the extract may not be highly potent in inhibiting cholinesterases, it still possesses some neuroprotective potential. Compounds with AChE and BChE inhibitory activity are often investigated for their role in supporting memory and cognitive function by increasing acetylcholine levels, making this extract a candidate for further studies on neurodegenerative disorders such as Alzheimer’s disease.

Studzinska-Sroka et al. [15] evaluated the neuroprotective potential of *E. prunastri* hexane, dichlormethane, acetone, methanolic, water, and methanol–water extracts using AChE, BChE, and tyrosinase assays. Among the tested extracts, only the water extract showed stronger inhibition of BChE compared to AChE. The results from our study also indicated higher BChE inhibition. Recent investigations have shown that BChE inhibitors or double BChE and AChE inhibitors possess better curative effects on Alzheimer disease, while at the same time, side effects are lower than those of specific AChE inhibitors [27]. Since AChE is predominantly localized in the CNS while BChE is more abundant in the peripheral system, the efficacy against both ChEs demonstrated for *E. prunastri* extract in our study is of utmost importance.

In terms of metabolic enzyme inhibition, the extract showed moderate α-amylase inhibitory activity with an IC_50_ value of 3.95 mg/mL. α-Amylase inhibition may help reduce the breakdown of starch, thereby reducing postprandial blood sugar spikes. The most striking activity was observed in α-glucosidase inhibition, with an IC_50_ value of 0.77 mg/mL. This significant inhibition of α-glucosidase, an enzyme critical for carbohydrate digestion and glucose absorption, suggests that the extract could play a role in regulating blood glucose levels, a potential benefit for managing diabetes and associated metabolic disorders. These properties, particularly the strong α-glucosidase inhibition, highlight the extract’s potential as an antidiabetic agent that can help with blood sugar control. Studzinska-Stroka et al. [15] showed that water extract, as well as one obtained using diluted methanol, had almost no inhibitory effect on the enzyme, while hexane extract rich in atranorin showed a significant inhibitory level, even at lower concentrations. In the same study, acarbose, used as a positive control, had a comparable effect to *E. prunastri* extracts (methanolic, hexane, acetone, and dichloromethane) tested at ten-times-lower concentrations. Amssayef et al. confirmed the antidiabetic potential of oakmoss in diabetic rats [28]. Namely, both single and oral repeated doses produced a significant reduction in blood glucose. Peng et al. reported that some flavonoids notably inhibited α-glucosidase as a result of conformational changes induced after their insertion into the active site of α-glucosidase [29].

The extract, however, did not exhibit tyrosinase inhibitory activity, as indicated by “na” (not active). Tyrosinase inhibitors are typically explored for their potential in skin-whitening products and treatments for hyperpigmentation disorders. The absence of tyrosinase inhibition implies that *E. prunastri* EtOAc extract may not be applicable for such cosmetic or dermatological uses.

Based on the structure–activity relationship, the observed enzyme inhibitory effect of the *E. prunastri* extract may be linked to the presence of chemical components previously reported in oakmoss. Shi et al. [30] reported that some flavonoids exhibited a significant AChE inhibitory effect and also had a synergistic effect with galanthamine. In another study by Hua et al. [31], significant amylase and glucosidase inhibitory effects were reported by a flavonoid. In addition, other compounds have also been reported by several authors [32,33,34,35] as enzyme inhibitors. In this sense, the enzyme inhibition data suggest that *E. prunastri* EtOAc extract holds promise for the development of therapeutics for neurodegenerative diseases and metabolic disorders, with its potent glucosidase inhibition being a key point of interest for further research in diabetes management.

### 2.3. Antioxidant Potential

Table 3 highlights the antioxidant properties of the EtOAc extract of *E. prunastri* and shows its effectiveness in several antioxidant tests. The extract demonstrated moderate phosphomolybdenum activity with an EC_50_ value of 4.60 mg/mL, suggesting a decent capacity for reducing power in a broad context. In the DPPH radical scavenging assay, it exhibited a strong antioxidant capacity with a value of 2.59 mg/mL, indicating a good ability to neutralize free radicals, which is valuable for counteracting oxidative stress.

The ABTS assay showed a notably activity with an IC_50_ value of 1.04 mg/mL; however, trolox possessed a great scavenging ability (IC_50_: 0.24 mg/mL). Similarly, the CUPRAC assay, measuring cupric-ion-reducing power, resulted in a significant value of 1.16 mg/mL. The FRAP assay, which measures ferric-reducing antioxidant power, gave a moderate result of 2.62 mg/mL, indicating that the extract has moderate effectiveness in terms of electron donation to neutralize oxidative agents.

Additionally, the extract’s metal-chelating activity was recorded with an IC_50_ value of 1.09 mg/mL. Metal chelation can help mitigate oxidative stress by reducing metal-catalyzed oxidation reactions, suggesting that *E. prunastri* extract could play a role in protecting biological systems from metal-induced oxidative damage.

*E. prunastri* EtOAc extract demonstrates a diverse and strong antioxidant profile, particularly with its high ABTS and CUPRAC values, indicating its promise as a natural antioxidant source for potential applications in preventing-oxidative stress-related disorders and extending the shelf-life of products in the food, cosmetic, and pharmaceutical industries.

Studzinska-Sroka et al. [36] investigated the antioxidant potential of acetone extracts of three lichen species, i.e., *E. prunastri*, *Cladonia uncialis*, and *Parmelia sulcata*. Compared to the other two species, *E. prunastri* had the weakest effect. Similarly, Aslan et al. pointed out that methanolic extracts of the lichens *Cladonia foliacea*, *Evernia divaricate*, and *E. prunastri* did not exhibit notable free radical scavenging activity [37].

### 2.4. Antibacterial Activity

According to available data in the literature, it is assumed that this is the first report of the antibacterial activity of *E. prunastri* EtOAc extract from the species collected in south Serbia. The results of antibacterial activity against important pathogenic bacteria are presented in Table 4. The MIC and MBC values varied from 0.0085 mg/mL to 0.14 mg/mL. All examined samples were more effective against Gram-positive (G^+^) bacteria than against Gram-negative (G^-^) bacteria. *Enterobacter cloacae* and *Escherichia coli* were the most sensitive among the G^+^ and G^-^ bacteria, with equal MICs and MBCs of 0.0085 mg/mL. The most resistant strain of bacteria was *Listeria monocytogenes*, with an MIC of 0.068 mg/mL and MBC of 0.14 mg/mL. The commercial drugs streptomycin and ampicillin were more active against all the tested bacteria. Such an effect was expected, since commercial drugs are single active compounds, while the extract presents a mixture of various compounds that may influence the final activity outcome.

Aoussar et al. showed that among tested lichen species, the strongest antibacterial activity was determined for *E. prunastri* methanolic extract, with MIC values ranging from 0.07 to 0.15 mg/mL against *Staphylococcus aureus* isolates from catheter-associated infections [23]. Also, Scherbakova et al., investigated the antimicrobial activity of various *E. prunastri* extracts (hexane, dichloromethane, and acetonitrile) against *S. aureus*, *Pseudomonas aeruginosa*, and *Escherichia coli* [38]. Both the dichloromethane and hexane extracts were active towards *S. aureus*, with MIC values of 4 and 21 μg/mL. Bekka-Hadjiand and Adjeroud-Abdellatif exhibited the high activity of *E. prunastri* methanolic extract against methicillin resistant *S. aureus* using a microdilution method [39]. In the study conducted by Aslan et al., methanolic extract showed greater antimicrobial capacity compared to antibiotics used as controls towards *Clavibacter michiganense*, *E. coli*, *P. syringae*, *Streptococcus pyogenes*, and *Xanthomonas campestris* [37]. A previous study also reported the antibacterial activity of an acetone–methanolic extract of *E. prunastri*, with MIC and MBC values ranging from 0.03125 mg/mL to 0.5 mg/mL, which was tested on four bacteria strains, including *S. aureus*, *E. coli*, *P. aeruginosa*, and MRSA strains. The extract published in this study was not active against *E. coli* [40], while our study showed the best activity of oakmoss EtOAc extract on *E. coli*. Such differences might be attributed to different solvents applied for the extraction. He et al. investigated the antimicrobial effects of natural compounds against *E. coli* and showed the most potent activity for a flavonoid [41]. 

While this study provides valuable insights into the bioactivity and chemical composition of *E. prunastri* extract, several limitations should be considered. The study relied on in vitro assays, and in vivo validation is necessary to confirm the therapeutic potential and safety of the extract. The mechanistic pathways underlying the observed bioactivities remain unclear, and further studies are needed to identify the specific targets involved. Furthermore, clinical trials are necessary to confirm the therapeutic relevance of the extract in human settings.

## 3. Materials and Methods

### 3.1. Collection of Material and Extraction

The lichen *Evernia prunastri* (L.) Ach. (Parmeliaceae) was collected from the wild, growing on mountain trees in Poljanica, Vranje, Serbia. The sample contained material from 25 lichens to ensure representative material for the investigations. Lichen was air-dried and reduced to a fine powder before the extraction procedure. The powdered lichen samples (~10 g) were extracted by stirring with 300 mL of EtOAc at 4 °C for 24 h. The extract was filtered through Whatman No. 4 paper. The residue was then re-extracted twice with additional portions (300 mL) of EtOAc. The combined extracts were evaporated at 30 °C (rotary evaporator Büchi R-210, Flawil, Switzerland) in order to remove the solvent.

### 3.2. Total Phenolic and Flavonoid Contents and LC-HRMS/MS

The total phenolic content was measured using the Folin–Ciocalteu reagent, following a method previously described in the literature. The results were expressed as milligrams of gallic acid equivalents (mg GAE) per gram of extract [42].

The total flavonoid content was determined using the AlCl_3_ method, with rutin as the reference standard. The results were expressed as milligrams of rutin equivalents (mg RE) per gram of extract [42].

LC-HRMS/MS (Thermo Scientific™ Vanquish™ Core HPLC system coupled to a Orbitrap Exploris 120 mass spectrometer, San Jose, CA, USA) was used to determine the metabolic profile of the extract [43]. The liquid chromatography system was equipped with an Hypersil GOLD™ C18 analytical column (50 × 2.1 mm, 1.9 μm particle size). The injection volume was 5 μL, and the flow rate was constant at 300 μL/min. The compounds of interest were eluted with ultrapure water + 0.1% formic acid (A) and acetonitrile (MS grade) + 0.1% formic acid (B): 5% B in the first min; 5–95% B from 1 to 10 min; 95% B from 10 to 12 min; 5% B until 15 min.

The Orbitrap Exploris 120 mass spectrometer was equipped with an ESI source operating in the negative ionization mode. Full-scan MS results were monitored from 100 to 1500 *m/z* with the Orbitrap resolution set to 60,000 FWHM, while data-dependent MS^2^ experiments were conducted at an Orbitrap resolution of 15,000 FWHM with the normalized collision energy of CID set to 35%. The dynamic exclusion time was set to 10 s with exclusion from a specific scan after 2 occurrences, and the intensity threshold was set to 1 × 10^5^. The identification of the phenolic compounds was carried out based on their chromatographic behaviour and MS and MS^2^ by comparison with standard compounds, when available, and data from the literature providing a tentative identification. Data acquisition was carried out with an Xcalibur^®^ data system (Thermo Finnigan, San Jose, CA, USA).

### 3.3. Biological Activities Evaluation

#### 3.3.1. Inhibitions of Enzymes Linked to Diabetes Mellitus Therapy

For the α-amylase inhibitory activity assay, the sample solution (50 µL, 0.5–5 mg/mL in methanol) was mixed with 50 μL of α-amylase solution (from porcine pancreas, EC 3.2.1.1, Sigma) in phosphate buffer (pH 6.9 with 6 mM sodium chloride) in a 96-well microplate and incubated for 10 min at 37 °C. After pre-incubation, the reaction was initiated by adding 50 μL of a 0.05% starch solution. A blank was prepared similarly but without α-amylase solution. The reaction mixture was incubated for another 10 min at 37 °C and stopped by adding 25 μL of 1 M HCl, followed by 100 μL of iodine–potassium iodide solution. The absorbances were measured at 630 nm. The negative control was prepared without the extract (only 50 µL of methanol). The blank absorbance was further subtracted from that of the sample, and the α-amylase inhibitory activity was expressed as IC_50_ value [42].

For the α-glucosidase inhibitory activity assay, the sample solution (50 µL, 0.5–5 mg/mL in methanol) was mixed with 50 µL of glutathione, 50 µL of α-glucosidase solution (from *Saccharomyces cerevisiae*, EC 3.2.1.20, Sigma), and 50 µL of PNPG (4-Nitrophenyl-α-D-glucopyranoside, Sigma) in phosphate buffer (pH 6.8) in a 96-well microplate and incubated for 15 min at 37 °C. A blank was prepared in the same manner, except that the α-glucosidase enzyme solution was omitted. The reaction was stopped by adding 50 µL of 0.2 M sodium carbonate. The absorbances of the sample and blank were measured at 400 nm. The negative control was prepared without the extract (only 50 µL of methanol). The absorbance of the blank was subtracted from the sample absorbance, and the α-glucosidase inhibitory activity was expressed as the IC_50_ value [42]. All chemicals mentioned as being from Sigma were obtained from St. Louis, MO, United States.

#### 3.3.2. Inhibitions of Enzymes Linked to Alzheimer’s and Parkinson’s Diseases Treatment

For the cholinesterase (ChE) inhibitory activity assay, the sample solution (100 µL, 0.5–5 mg/mL in methanol) was mixed with DTNB (5,5-dithio-bis(2-nitrobenzoic) acid, Sigma, St. Louis, MO, USA) (125 µL) and AChE (acetylcholinesterase (Electric ell acetylcholinesterase, Type-VI-S, EC 3.1.1.7, Sigma)) or BChE (butyrylcholinesterase (horse serum butyrylcholinesterase, EC 3.1.1.8, Sigma)) solution (25 μL) in Tris–HCl buffer (pH 8.0) in a 96-well microplate and incubated for 15 min at 25 °C. The reaction was then initiated with the addition of acetylthiocholine iodide (ATCI, Sigma) or butyrylthiocholine chloride (BTCl, Sigma) (25 μL). Similarly, a blank was prepared by adding the sample solution to all reaction reagents without the enzyme (AChE or BChE) solution. The sample and blank absorbances were read at 405 nm after 10 min incubation at 25 °C. The negative control was prepared without the extract (only 100 µL of methanol). The absorbance of the blank was subtracted from that of the sample, and the cholinesterase inhibitory activity was expressed as the IC_50_ value [42].

For the tyrosinase inhibitory activity assay, the sample solution (50 µL, 0.5–5 mg/mL in methanol) was mixed with tyrosinase solution (40 μL, Sigma) and phosphate buffer (100 μL, pH 6.8) in a 96-well microplate and incubated for 15 min at 25 °C. The reaction was then initiated with the addition of L-DOPA (40 μL, Sigma). Similarly, a blank was prepared by adding the sample solution to all reaction reagents without the enzyme (tyrosinase) solution. The sample and blank absorbances were read at 492 nm after a 10 min incubation at 25 °C. The negative control was prepared without the extract (only 50 µL of methanol). The absorbance of the blank was subtracted from that of the sample, and the tyrosinase inhibitory activity was expressed as the IC_50_ value [42]. All chemicals mentioned as being from Sigma were obtained from St. Louis, MO, United States.

#### 3.3.3. Antioxidant Activity Assays

Antioxidant (DPPH and ABTS radical scavenging, reducing power (CUPRAC and FRAP), phosphomolybdenum and metal chelating (ferrozine method)) activity was determined using the methods previously described by Uysal et al. [42]. DPPH, ABTS, and metal chelating were expressed as the IC_50_ value, whereas CUPRAC, FRAP, and phosphomolybdenum were evaluated as the EC_50_ (effective concentration at absorbance 0.5) value.

#### 3.3.4. Antibacterial Activity Assay

For the antibacterial activity, the following G^−^ bacteria were used: *Escherichia coli* (ATCC 35210), *Pseudomonas aeruginosa* (ATCC 27853), *Salmonella typhimurium* (ATCC 13311), *Listeria monocytogenes* (NCTC 7973), and *Enterobacter cloacae* (human isolate); and the following G^+^ bacteria were used: *Bacillus cereus* (clinical isolate), *Micrococcus flavus* (ATCC 10240), and *Staphylococcus aureus* (ATCC 6538). The antibacterial assay was carried out by the microdilution method.

The bacterial cell suspension was adjusted with sterile physiological saline to a concentration of approximately 1.0 × 10^5^ CFU in a final volume of 100 μL per well. The extract for testing was dissolved 5% DMSO (1 mg/mL) and added in Triptic Soy broth (TSB) medium (100 μL) with bacterial inoculum (1.0 × 10^5^ CFU per well) to achieve the wanted concentration (serial dilution range of concentrations applied was 0.004375 mg/mL–0.28 mg/mL, dilution factors 1, 1:2, 1:4, 1:8, 1:16, 1:32, and 1:64). The microplates were incubated at rotary shaker (160 rpm) for 24 h at 37° C. The following day, 30 μL of 0.2 mg/mL solution of INT (p-iodonitrotetrazolium violet) was added, and the plates were returned to the incubator for at least half an hour to ensure adequate color reaction [44,45]. Growth inhibition was indicated by a clear solution or a noticeable reduction in color intensity. The lowest concentrations that showed no visible growth under a binocular microscope were considered the concentrations that completely inhibited bacterial growth (minimum inhibitory concentrations, MICs). To determine the minimum bactericidal concentrations (MBCs), serial sub-cultures of 2 μL were inoculated into microtiter plates containing 100 μL of broth per well, followed by incubation for 24 h. The MBC was defined as the lowest concentration with no visible growth, representing a 99.5% reduction in the original inoculum. The optical density (OD) of each well was measured at a wavelength of 655 nm using Microplate Manager 4.0 (Bio-Rad Laboratories), and the results were compared to a blank and a positive control. Streptomycin and ampicillin (1 mg/mL in sterile physiological saline) were used as positive controls, while 5% DMSO was used as a negative control. Three independent experiments were conducted in triplicate.

### 3.4. Statistical Analysis

Three samples were used, and all the assays were carried out in triplicate. The results are expressed as mean values and standard deviation (SD) and were analyzed using one-way analysis of variance (ANOVA) followed by Tukey’s HSD test with α = 0.05. This analysis was carried out using SPSSv.18.0.

## 4. Conclusions

The EtOAc extract of *E. prunastri* demonstrated a broad spectrum of biological activities, highlighting its potential as a valuable functional food ingredient with multiple health benefits. The extract exhibited significant inhibitory effects on enzymes linked to diabetes mellitus (α-amylase and α-glucosidase) and neurodegenerative diseases (acetylcholinesterase and butyrylcholinesterase), suggesting potential therapeutic applications in managing metabolic and cognitive disorders. Notably, the extract showed potent α-glucosidase inhibition, which could contribute to blood glucose regulation and support diabetes management. The antioxidant activity of the extract, as evidenced by the assay results, underscores its ability to mitigate oxidative stress, a factor implicated in aging and various chronic diseases. The antibacterial activity against both G^+^ and G^-^ bacteria further emphasizes its potential role in combating microbial infections, aligning with the global demand for novel antimicrobial agents to address rising antibiotic resistance. Although the extract did not demonstrate tyrosinase inhibitory activity, its efficacy in other enzyme inhibition assays and antioxidant tests positions it as a multifaceted bioactive agent. This study provides compelling evidence that *E. prunastri* can be classified as a functional food with numerous pharmacological effects, supporting its inclusion in dietary interventions aimed at promoting health and preventing disease. The promising bioactivity demonstrated by *E. prunastri* extract opens avenues for future research and application, particularly in the development of functional foods and therapeutic agents. Future studies should focus on validating the therapeutic potential of the extract in vivo to assess its efficacy and safety, providing a clearer understanding of its pharmacokinetics and bioavailability. Additionally, exploring the synergistic effects of the extract’s bioactive compounds, as well as investigating their molecular mechanisms of action, could offer valuable insights into how these compounds interact at the cellular level. Clinical trials in human subjects are essential to determine the clinical relevance of the extract for conditions like diabetes, Alzheimer’s disease, and bacterial infections. Finally, standardizing the extraction process and exploring the potential variations in chemical composition due to environmental factors will be important for ensuring consistent therapeutic efficacy. *E. prunastri* represents a promising natural resource with vast therapeutic potential, warranting further exploration in the context of modern medicine.

## Figures and Tables

**Table 1:** Total phenolic and flavonoid contents, identified compound names, retention times (Rt, min), molecular formulas, MS data (calculated and exact masses, as well as mean mass accuracy—Δ mDa), and major MS^2^ fragment ions of the compounds present in the EtOAc extract of *E. prunastri*.

Sample			Total Phenolic Content (mgGAE/g Extract)				Total Flavonoid Content (mgRE/g Extract)
*E. prunastri* EtOAc extract			22.11 ± 1.24				13.64 ± 0.30
**No**	**Compound Name**	***t*_R_, min**	**Molecular Formula, [M–H]^−^**	**Calculated Mass, *m*/*z***	**Exact Mass, *m*/*z***	**Δ ppm**	**MS^2^ Fragments, (% Base Peak)**
1	Orsellinic acid	5.58	C_8_H_7_O_4_^−^	167.03500	167.03504	−0.22	123.04523(100), 167.03503(55)
2	3,7-Dihydroxy-1-methyldibenzofuran-2,9-dicarboxylic acid	6.64	C_15_H_9_O_7_^−^	301.03540	301.03544	−0.11	213.05565(100), 257.04565(6)
3	Salazinic acid	7.14	C_18_H_11_O_10_^−^	387.03577	387.03534	1.12	121.02954(48), 227.03505(75), 241.05061(38), 243.02998(69), 269.04541(100), 343.04575(30)
4	Hematommic acid	7.23	C_9_H_7_O_5_^−^	195.02990	195.02986	0.20	123.04517(91), 149.02452(12), 151.04008(100), 177.01930(3), 195.02986(80)
5	3-Hydroxy-4-(methoxycarbonyl)-5-methylphenyl 3,4,6-trihydroxy-2-methylbenzoate	7.92	C_17_H_15_O_8_^−^	347.07724	347.07679	1.30	123.04523(13), 124.04861(8), 149.02451(48), 150.02788(40), 167.03505(100), 168.03845(83)
6	Lecanoric acid	7.98	C_16_H_13_O_7_^−^	317.06668	317.06660	0.25	123.04522(10), 149.02452(16), 167.03502(100)
7	Parmosidone B	8.15	C_17_H_13_O_9_^−^	361.05651	361.05608	1.17	109.02952(7), 135.00880(18), 153.01935(100), 197.00917(41)
8	Consalazinic acid	8.50	C_18_H_13_O_10_^−^	389.05142	389.05147	−0.12	123.04518(10), 149.02446(100), 167.03499(88)
9	Conechinocarpic acid	8.62	C_20_H_19_O_9_^−^	403.10346	403.10284	1.54	149.02449(39), 177.01935(91), 193.01427(100), 218.05847(42), 225.04048(46), 246.05338(45)
10	Squamatic acid	9.10	C_19_H_17_O_9_^−^	389.08781	389.08714	1.72	105.03460(10), 149.02441(25), 163.04007(100), 193.01421(56), 195.06619(6)
11	Evernic acid	9.12	C_17_H_15_O_7_^−^	331.08238	331.08197	1.24	123.04512(11), 149.02443(66), 167.03496(100)
12	Hydroxyphysodic acid	9.23	C_26_H_29_O_9_^−^	485.18171	485.18141	0.62	235.09755(83), 261.07687(39), 397.20251(23), 399.18118(100), 423.18124(33), 441.19193(25)
13	Chloroevernic acid	9.23	C_17_H_14_ClO_7_^−^	365.04345	365.04289	1.53	123.04517(12), 149.02443(54), 167.03494(100)
14	Evernin	9.67	C_19_H_19_O_7_^−^	359.11363	359.11291	2.00	137.06085(13), 167.03502(18), 177.01938(36), 181.05066(100), 195.06618(10), 341.02997(88)
15	Cristiferide B	9.69	C_19_H_16_ClO_9_^−^	423.04883	423.04854	0.69	138.99562(29), 163.04016(52), 182.98546(74), 226.97523(100)
16	Physodic acid	9.73	C_26_H_29_O_8_^−^	469.18679	469.18654	0.53	283.13394(30), 357.20715(14), 381.20715(100), 383.18631(84), 425.19684(55)
17	Pseudoplacodiolic acid	9.83	C_19_H_19_O_8_^−^	375.10854	375.10822	0.85	259.06158(20), 299.09250(59), 301.07175(40), 343.08218(62), 375.10843(100)
18	Dimethylpannaric acid	10.41	C_18_H_15_O_7_^−^	343.08233	343.08212	0.60	259.06119(65), 299.09259(12), 328.05887(100), 343.08212(19)
19	Atranorin	10.43	C_19_H_17_O_8_^−^	373.09289	373.09281	0.23	163.04008(100), 177.0193(46), 181.05074(4), 195.06606(4), 329.06677(6), 355.04614(11)
20	Chloroatranorin	10.74	C_19_H_16_ClO_8_^−^	407.05392	407.05362	0.74	138.99571(7), 163.04013(100), 166.99066(15), 195.06592(5), 210.98039(100)

GAE: gallic acid equivalent; RE: rutin equivalent.

**Table 2:** Enzyme inhibitory properties of *E. prunastri* (IC_50_ (mg/mL) (mean ± SD).

Samples	AChE	BChE	Amylase	Glucosidase	Tyrosinase
*E. prunastri* EtOAc extract	1.26 ± 0.03 ^b^	1.70 ± 0.09 ^b^	3.95 ± 0.47 ^b^	1.01 ± 0.01 ^b^	na
Galanthamine	0.003 ± 0.0001 ^a^	0.004 ± 0.0001 ^a^	-	-	-
Acarboıse	-	-	1.29 ± 0.02 ^a^	0.77 ± 0.03 ^a^	-
Kojic acid	-	-	-	-	0.09 ± 0.01

na: not active. In each column, different letters mean significant differences between the samples (*p* < 0.05).

**Table 3:** Antioxidant properties of *E. prunastri* (IC_50_ (mg/mL) (mean ± SD).

Sample	Phosphomolybdenum	DPPH	ABTS	CUPRAC	FRAP	Metal Chelating Activity
*E. prunastri* EtOAc extract	4.60 ± 0.16 ^b^	2.59 ± 0.19 ^b^	1.04 ± 0.01 ^b^	1.16 ± 0.03 ^b^	2.62 ± 0.07 ^b^	1.09 ± 0.05 ^b^
Trolox	0.56 ± 0.04 ^a^	0.11 ± 0.01 ^a^	0.24 ± 0.01 ^a^	0.15 ± 0.01 ^a^	0.09 ± 0.01 ^a^	-
EDTA	-	-	-	-	-	0.20 ± 0.01 ^a^

In each column, different letters mean significant differences between the samples (*p* < 0.05).

**Table 4:** Antibacterial activity of *E. prunaastri* EtOAc extract (mg/mL).

	*E. prunastri* EtOAc		Streptomycin		Ampicillin	
Bacteria	MIC	MBC	MIC	MBC	MIC	MBC
*S. aureus*	0.034 ^c^	0.034 ^c^	0.000367 ^b^	0.000733 ^b^	0.000217 ^a^	0.000433 ^a^
*B. cereus*	0.00875 ^c^	0.017 ^c^	0.000303 ^b^	0.000607 ^b^	0.00013 ^a^	0.000267 ^a^
*M. flavus*	0.017 ^c^	0.14 ^c^	0.00035 ^a^	0.0007 ^a^	0.000433 ^b^	0.000867 ^b^
*L. monocytogenes*	0.068 ^c^	0.14 ^c^	0.013333 ^b^	0.026667 ^b^	0.00014 ^a^	0.000282 ^a^
*P. aeruginosa*	0.00875 ^c^	0.017 ^c^	0.000367 ^b^	0.000733 ^b^	0.000135 ^a^	0.000275 ^a^
*E. cloacae*	0.00875 ^c^	0.00875 ^c^	0.000367 ^b^	0.000733 ^b^	0.000217 ^a^	0.000433 ^a^
*S. typhimurium*	0.017 ^b^	0.017 ^b^	0.00045 ^a^	0.0009 ^a^	0.00045 ^a^	0.000917 ^a^
*E. coli*	0.00875 ^c^	0.00875 ^c^	0.000433 ^b^	0.000867 ^b^	0.000145 ^a^	0.00029 ^a^

In each line, different letters mean significant differences between the MICs and MBCs of the extracts and positive controls (*p* < 0.05).

## Data Availability

The original contributions presented in this study are included in the article. Further inquiries can be directed to the corresponding authors.
